# DHCR7 Expression Predicts Poor Outcomes and Mortality from Sepsis

**DOI:** 10.21203/rs.3.rs-2500497/v1

**Published:** 2023-01-30

**Authors:** Faheem W. Guirgis, Vinitha Jacob, Dongyuan Wu, Morgan Henson, Kimberly Daly-Crews, Charlotte Hopson, Lauren Page Black, Elizabeth L. DeVos, Dawoud Sulaiman, Guillaume Labilloy, Todd M. Brusko, Jordan A. Shavit, Andrew Bertrand, Matthew Feldhammer, Brett Baskovich, Kiley Graim, Susmita Datta, Srinivasa T. Reddy

**Affiliations:** University of Florida College of Medicine – Jacksonville; University of Michigan College of Medicine; University of Florida; University of Florida College of Medicine – Jacksonville; University of Florida College of Medicine – Jacksonville; University of Florida College of Medicine – Jacksonville; University of Florida College of Medicine – Jacksonville; University of Florida College of Medicine – Jacksonville; David Geffen School of Medicine at UCLA; UF Health Jacksonville Center for Data Solutions; University of Florida Diabetes Institute; University of Michigan School of Medicine Ann Arbor Michigan; University of Florida College of Medicine – Jacksonville; University of Florida College of Medicine – Jacksonville; Mt. Sinai School of Medicine; University of Florida; University of Florida; David Geffen School of Medicine at UCLA

**Keywords:** sepsis, lipids, genetics, transcriptomics, zebrafish

## Abstract

**Objective:**

Sepsis patients experience poor outcomes including chronic critical illness (CCI) or early death (within 14 days). We investigated lipid metabolic gene expression differences by outcome to discover therapeutic targets.

**Design:**

Secondary analysis of samples from prospectively enrolled sepsis patients and a zebrafish sepsis model for drug discovery.

**Setting:**

Emergency department or ICU at an urban teaching hospital.

**Patients:**

Sepsis patients presenting within 24 hours.

**Methods:**

Enrollment samples from sepsis patients were analyzed. Clinical data and cholesterol levels were recorded. Leukocytes were processed for RNA sequencing (RNA-seq) and reverse transcriptase polymerase chain reaction (RT-qPCR). A lipopolysaccharide (LPS) zebrafish sepsis model was used for confirmation of human transcriptomic findings and drug discovery.

**Measurements and Main Results:**

There were 96 samples in the derivation (76 sepsis, 20 controls) and 52 in the validation cohort (sepsis only). The cholesterol metabolism gene 7-Dehydrocholesterol Reductase *(DHCR7)* was significantly upregulated in both derivation and validation cohorts in poor outcome sepsis compared to rapid recovery patients and in 90-day non-survivors (validation only) and validated using RT-qPCR analysis. Our zebrafish sepsis model showed upregulation of *dhcr7* and several of the same lipid genes upregulated in poor outcome human sepsis *(dhcr24, sqlea, cyp51, msmol, ldlra)* compared to controls. We then tested six lipid-based drugs in the zebrafish sepsis model. Of these, only the *Dhcr7* inhibitor AY9944 completely rescued zebrafish from LPS death in a model with 100% lethality.

**Conclusions:**

*DHCR7*, an important cholesterol metabolism gene, was upregulated in poor outcome sepsis patients warranting external validation. This pathway may serve as a potential therapeutic target to improve sepsis outcomes.

## Introduction

Sepsis is a dysregulated response to infection and is the costliest reason for hospital admission world-wide.^1–4^ It occurs when a systemic infection results in a dysregulated immune response that leads to organ failure and potentially death.^3^ Survivors of sepsis are frequently left with reduced quality of life physical function, and long-term survival.^5–7^ Our group has defined and described clinically relevant outcomes that include early death (death within the first 14 days of sepsis), chronic critical illness (CCI, ICU stay >14 days with organ failure), and rapid recovery (neither early death nor CCI). CCI patients frequently develop the persistent inflammation immunosuppression and catabolism syndrome (PICS), characterized by impaired physical function and one-year mortality rates over 40%.^7,8^

We and others have described the protective role of lipids and lipoproteins in sepsis.^9–12^ High density lipoprotein (HDL) has antioxidant and anti-inflammatory proteins (paraoxonase-1 and apolipoprotein A-I) that protect against lipid oxidation, prevent inflammatory cell activation and chemotaxis, bind and clear bacterial toxins, and downregulate inflammatory pathways.^9–17^ Similarly low density lipoprotein (LDL) may play a role in bacterial endotoxin clearance via the LDL receptor, particularly in gram negative sepsis, with proprotein convertase subtilisin/kexin type 9 (PCSK9) playing an important regulatory role.^18–21^ However, dysregulated lipid metabolism occurs in sepsis leading to HDL’s conversion to dysfunctional and pro-inflammatory HDL (Dys-HDL) that may play a role in organ failure progression, and the pathogenesis of CCI, and PICS.^22–24^

Recent studies have shed some light on the genetic underpinnings of lipid metabolism in sepsis. A UK Biobank study identified an important link between genetically determined HDL-C levels and decreased risk of hospitalizations for infectious disease, lower odds of outpatient antibiotic usage, and reduced risk of mortality from sepsis.^25^ LDL-C and triglycerides levels did not confer the same risk reduction.^25^ However, the UK Biobank population were of homogenous ancestry. Another study identified that a rare missense variant in the cholesteryl ester transfer protein *(CETP)* gene (lowers HDL-C levels) was linked with reductions in HDL-C during sepsis.^26^ Carriers of this risk allele had more severe organ failure and reduced 28-day survival.

Genetic studies of diverse cohorts are needed to understand the role of dysregulated lipid and lipoprotein metabolism in sepsis. This study sought to leverage a diverse prospective cohort of sepsis patients to investigate transcriptional profiles relevant to lipid metabolism in sepsis and associate these differences with relevant outcomes. The primary objective was to analyze leukocyte gene expression patterns of sepsis patients by clinical outcomes by performing both an unbiased RNA-seq analysis and a focused analysis of relevant lipid metabolism genes (47 genes selected *a priori)*. Results were corroborated in a zebrafish model of endotoxemia, which further allowed the functional testing of relevant genes. Zebrafish were selected as they are vertebrates that share many anatomic and physiologic similarities with humans, most aspects of the immune response, and nearly all elements of lipid and lipoprotein metabolism.^27–29^ These investigations may aid the identification of lipid metabolic pathways that are critical for regulating the response to sepsis and identifying new potential therapies.

## Methods

### Design

We performed a secondary analysis of transcriptomic data from four prospective studies of sepsis patients enrolled between November 2016 and July 2022 from the emergency department at UF Health Jacksonville. All human studies were approved by the University of Florida Institutional Review Board (IRB-01, approved through 01/06/2023) and registered with clinicaltrials.gov(NCT02934997; NCT04576819; NCT03405870). STROBE guidelines for observational studies were followed.^30^ Approval for all zebrafish work was granted by the Institutional Animal Care & Use Committee (IACUC protocol PROOOOl 0679; expiration date 3/10/2025) at The University of Michigan (Animal Welfare Assurance Number on file with the NIH Office of Laboratory Animal Welfare is A3114–01).

### Patient Selection and Enrollment

Patients enrolled in the UF JAX Sepsis Biobank were considered eligible for inclusion after IRB approval. UF Health Jacksonville emergency department patients meeting Sepsis-3 criteria were identified prospectively by trained research coordinators or providers within 24 hours of sepsis recognition.^3^ Patient enrollment occurred seven days per week between the hours of 8 am and 10 pm. Patients from three observational studies and one ongoing clinical trial (LIPid Intensive Drug therapy for Sepsis Pilot, LIPIDS-P) were included in this analysis.^31,32^ Exclusion criteria were overall similar to prior studies.^31,32^

### Data Collection

All clinical and laboratory data were reviewed and entered into a Research Electronic Data Capture (REDCap) database by trained research coordinators. Prospectively collected data included demographics, place of residence, source of infection, and comorbidities. Clinical variables included triage and enrollment vital signs, SOFA score, timing of antibiotics, volume of intravenous fluids, vasopressor use and duration, and mechanical ventilation use and duration. Hospital length of stay (LOS), and ICU LOS were documented.

### Clinical Outcomes and Adjudication

The primary outcome was one of three categories: 1) early death (within 2 weeks of sepsis onset), 2) CCI (total ICU stay >14 days with organ dysfunction or total ICU <14 days but discharged to long-term acute care, another hospital, or hospice), or 3) rapid recovery (all others).^8^ Group adjudication by at least two clinician investigators was performed for the sepsis diagnosis, primary outcomes, primary and secondary source of infection, culture positivity and hospital disposition during sepsis adjudication meetings.^33^ Discrepancies were resolved by the inclusion of a third clinician investigator. The social security death index was used to determine mortality for patients lost to follow up. Twenty-eight and ninety-day mortality were also recorded.

### Blood Sampling, RNA-seq and RT-qPCR Analysis

Blood was drawn at the time of enrollment and within 24 hours of sepsis recognition and prior to any clinical trial drug administration. Clinical laboratory testing included cholesterol levels, and sequential organ failure assessment (SOFA) score laboratory measures including platelets, creatinine, and total bilirubin levels. Serum total cholesterol, HDL-C, and triglyceride levels were directly measured from serum samples. LDL-C was calculated using the Friedewald formula.^34^ RNA-seq was performed using the Illumina NextSeq 550 system (San Diego, CA). RT-qPCR was performed using Bio-Rad iQ SYBR Green Supermix (Cat# 1708882). For details on RNA-seq and RT-qPCR refer to Supplemental Material 1.

### Zebrafish experiments

Zebrafish were maintained according to protocols approved by the University of Michigan

Animal Care and Use Committee. All wild-type fish were a hybrid line generated by crossing AB and TL fish acquired from the Zebrafish International Resource Center. For details on cholesterol metabolism drug experiments, RT-qPCR, and RNA-seq analysis refer to Supplemental Material 1.

### Data Analysis

#### Univariate Comparisons

Presenting vital signs, cholesterol levels, demographic information, clinical features, and clinical management data across the outcome groups and by mortality (28 and 90 day) were analyzed. We calculated medians and interquartile ranges for continuous variables and counts and proportions for categorical variables. To test for differences among outcome groups, we ran the Shapiro-Wilkes test of normality for each of the continuous variables. Only age was found to be normally distributed. Age was also found to have homogeneity of variances, per Bartlett’s test, thereby meeting the requirements to use an ANOVA procedure.^40^ For all other continuous variables, we used the non-parametric Kruskal-Wallis procedure. We used Fisher’s Exact test to compare differences in categorical variables. We conducted a total of 28 tests comparing differences with the outcome group variable (see Tables 1 and 2), then applied Bonferroni adjustment to proportionally correct our presented p-values. Analysis and calculations were completed in R (version 4.1.2; Vienna, Austria) using statistical tests from the Stats package.

### Differential Expression Data Analysis

For data alignment, gene counts were obtained by aligning reads to the hg38 genome (GRCh38.p11) using STAR^36^ (v.2.7.9a) and featureCounts^37^ (v.2.0.3). We had two steps of analysis for the differential expression analysis: derivation and validation. We ensured a similar distribution of clinical outcomes across derivation and validation sets to detect differential expression patterns by outcome. To simplify the differential expression analysis, we combined early death and CCI patients into a “poor outcomes” group and compared them to rapid recovery patients who had more favorable outcomes. In a similar manner, we also performed a differential expression analysis by 90-day mortality. Twenty control samples were analyzed with the sepsis samples in the derivation set to compare gene expression patterns between the broader cohort of sepsis patients to healthy controls. The same differentially expressed genes detection protocol was used for both the derivation and validation steps of analysis. We included samples from two duplicate patients (both included in the validation set) enrolled in the study during two different sepsis episodes, over one year apart. Data were analyzed with and without these two additional patient encounters; their inclusion did not change the significant differentially expressed genes and so these encounters were included in the final results. In brief, the differential expression analysis was performed using DESeq2^38^ in R (version 4.0.5; Vienna, Austria). Gene counts were modeled with a negative binomial generalized linear model and adjusted for batch effects. Wald tests were conducted for the pairwise comparisons. We identified genes with adjusted p-values (i.e., p-values after false discovery rate correction) less than 0.05 as the differentially expressed genes. We focused our analysis on a set of 47 prespecified lipid metabolism genes (Supplemental Table 1).

## Results

The analysis included 128 sepsis patient encounters and 20 healthy controls. The derivation cohort included 96 patients and controls (12 early death, 13 CCI, 51 rapid recovery, and 20 controls) and the validation cohort had 52 patients (six early death, eight CCI, and 38 rapid recovery). For sepsis patients, presenting vital signs were similar by outcomes. Distribution of comorbidities across the outcome groups were similar (Table 1). Initial LDL-C levels were significantly lower for patients with early death or CCI compared to rapid recovery patients. Total cholesterol, HDL-C, and triglyceride levels were not statistically significantly different between groups. CCI patients were significantly older (median 72 years) than early death (median 61.5 years) or rapid recovery (median 60 years). Median SOFA and APACHE II scores were significantly higher for CCI (11, 18, respectively) and early death (10, 21, respectively) compared to rapid recovery (5, 13, respectively) patients. There was a higher proportion of septic shock patients in the early death and CCI groups compared to rapid recovery. The most common source of infection was pulmonary (27%), urinary tract (25%), and multiple sources of infection (17%). There were no significant differences in patient management characteristics (Table 2).

For the differential expression analysis, the derivation cohort had 96 single-end sequencing samples, including 12 early death, 13 CCI, 51 rapid recovery, and 20 healthy control patient samples. The validation cohort had 58 paired-end sequencing samples of sepsis patients, including eight early death, 12 CCI, and 38 rapid recovery. Patients included in the derivation cohort had a similar age, gender, and race distribution compared to patients in the validation set. With the exception of triglycerides, presenting cholesterol and lipid levels were similar between derivation and validation cohorts. They also had similar Apache II and SOFA scores, proportions of shock patients, and clinical management (Supplemental Tables 2 and 3).

**Figure 1:**
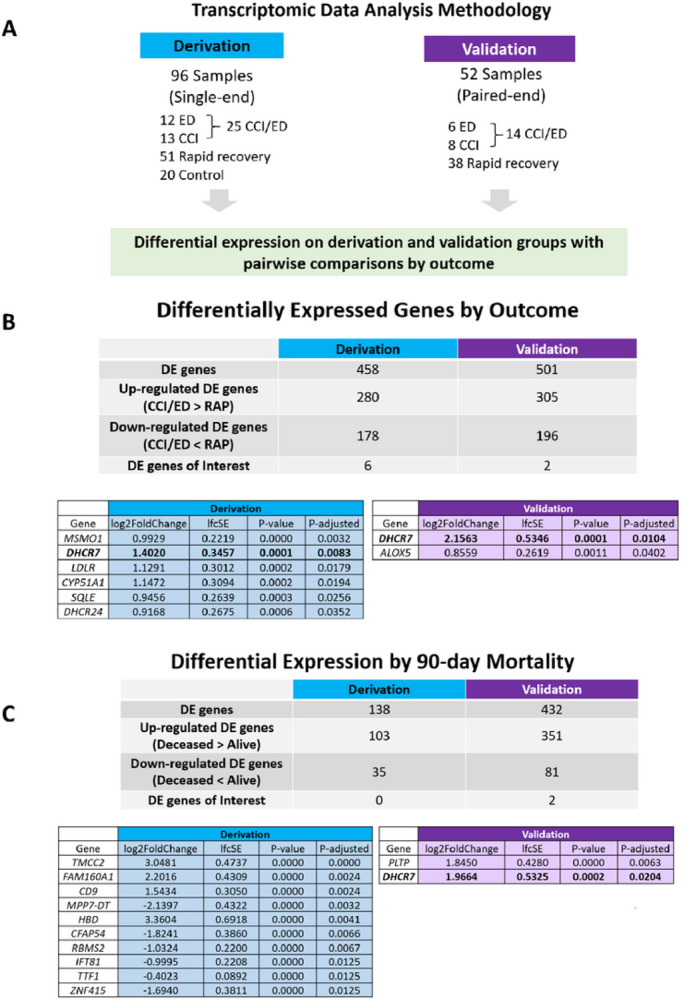


**Figure 2:**
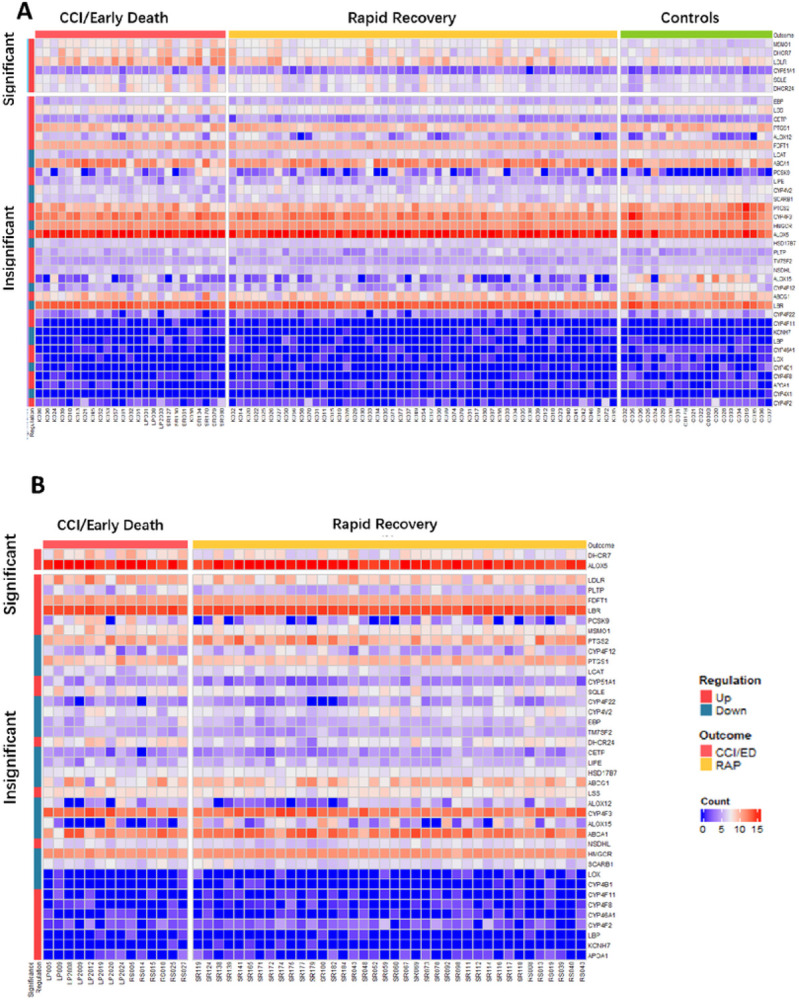


Figure 1 depicts the workflow for RNA-seq data analysis (1A) and significantly differentially expressed genes for the derivation and validation cohorts (1B) and by 90-day mortality (1C). In the derivation cohort, 458 of 39,372 genes were differentially expressed by patient outcome, including six of the 47 lipid metabolism genes of interest. In the validation cohort, 501 of 36,585 genes were identified as differentially expressed genes, including 2 lipid genes of interest. Of the 47 lipid metabolism genes of interest, there were 6 significant genes identified in the derivation cohort *(CYP51A1, DHCR24, DHCR7, MSMO1, SQLE*, and *LDLR*, and 2 genes identified in the validation cohort (*DHCR7*and *ALOX5)*. All of these genes were upregulated in early death/CCI patients when compared to rapid recovery patients. Figure 2 displays heatmaps of differentially expressed genes for derivation and validation cohorts. Five of the significant derivation cohort genes encode enzymes that catalyze critical steps in the biosynthesis of cholesterol *(CYP51A1, DHCR24, DHCR7, MSMO1, SQLE). CYP51A1* is critical for cholesterol synthesis, steroid synthesis, and drug metabolism.^41^ *LDLR* encodes the LDL receptor which endocytoses LDL-C from circulation.^42^ Both significant genes from the validation cohort were upregulated in CCI/early death patients compared to rapid recovery. *ALOX5*is the critical enzyme for the generation of all leukotrienes, potent mediators of inflammation.^43^ The only gene identified to be significantly upregulated in both cohorts was *DHCR7*. All the differentially expressed genes for derivation and validation cohorts are presented in Supplemental Material 2.

We performed a differential expression analysis by 90-day mortality. None of the lipid metabolism genes of interest were detected in the derivation cohort. However, *DHCR7* and *PLTP* were detected and upregulated in the validation cohort (Fig. 1). PLTP encodes a protein that is important for cholesterol and LPS clearance, and transfers phospholipids from triglyceride-rich lipoproteins. It also helps to regulate HDL size and is involved in cholesterol and LPS clearance.^24^

We next examined gene expression in sepsis patients and healthy controls by RT-qPCR. Based on availability of total RNA, we picked 10 CCI, 12 early death, 12 rapid recovery patients and 11 healthy controls for RT-qPCR. Demographics of patients included in RT-qPCR are presented in Supplemental Table 4. Five of the six genes *(LDLR, DHCR24, DHCR7, MSMO1, SQLE)* identified in the RNA-seq analysis were significantly upregulated in comparison to controls, while *CYP51A1* was not (Supplemental Fig. 1).

**Figure 3:**
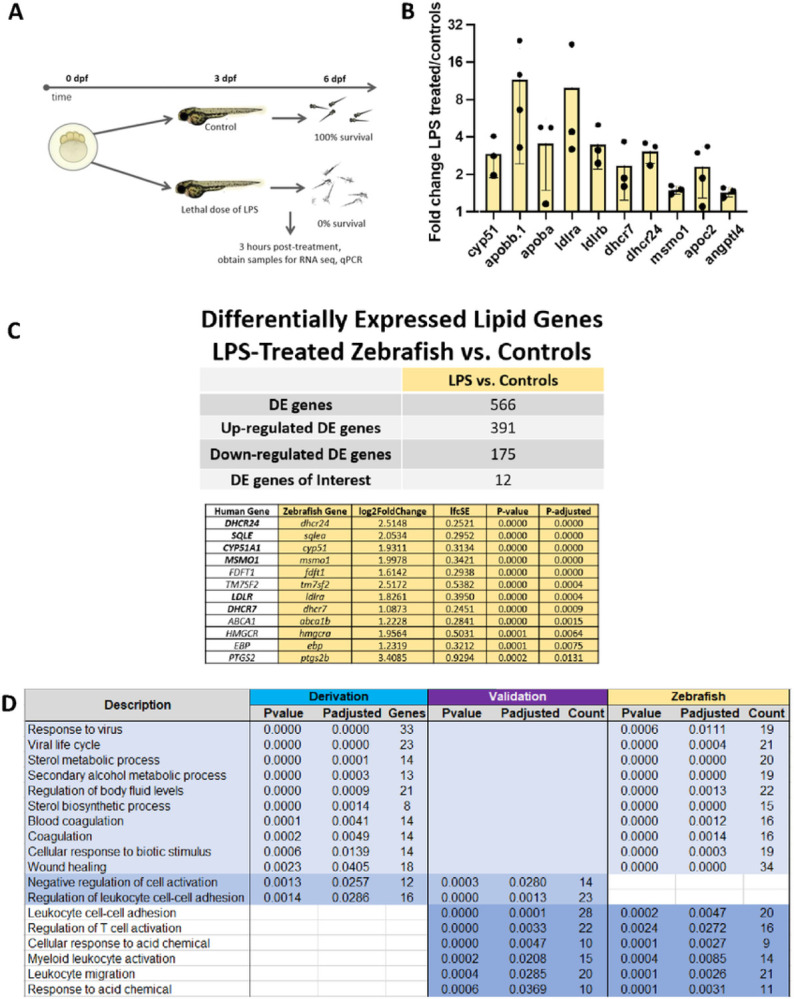


Workflow for zebrafish experiments with LPS versus controls is depicted in Fig. 3A RT-qPCR of cholesterol related genes showed upregulation of genes for the LDL receptor *(ldlra, ldlrb), dhcr7, dhcr24, msmo1, andcyp51* in LPS-treated zebrafish compared to controls (Fig. 3B). Differential expression analysis of RNA-seq data from three LPS treated zebrafish and three controls identified 12 lipid metabolism genes that were upregulated in LPS-treated zebrafish compared to controls (Fig. 3C). Notably, 6 of the genes *(dhcr7 dhcr24, sqlea, cyp51, msmo1*, and *ldlra)* were also upregulated in CCI/early death sepsis patients in the derivation cohort, as was *dhcr7* in the validation cohort. Overlap of significantly differentially expressed genes between derivation, validation, and zebrafish groups is depicted in Fig. 3D. Gene primers for zebrafish experiments are noted in Supplemental Material 3.

**Figure 4:**
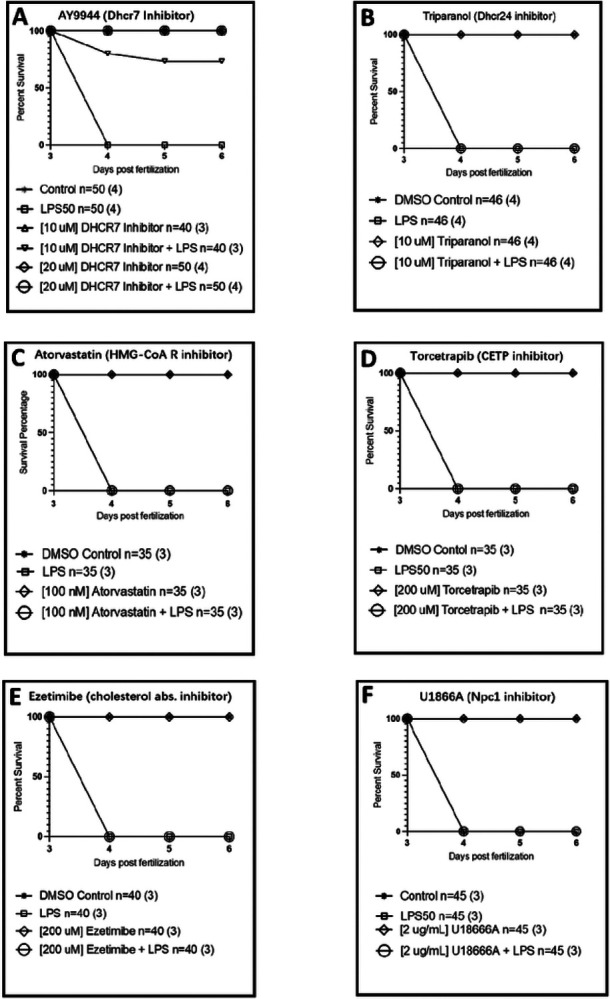


We tested several cholesterol metabolism drugs in our zebrafish model including AY9944 *(Dhcr7* inhibitor), triparanol *(Dhcr24* inhibitor), atorvastatin (HMG-CoA reductase inhibitor), torcetrapib (CETP inhibitor), and ezetimibe (cholesterol absorption inhibitor). Results of all zebrafish drug experiments are displayed in Fig. 4. Varying concentrations of each drug were administered at 3 dpf (days post fertilization) with or without a dose of LPS that caused complete lethality by 4 dpf. For AY9944 *(Dhcr7 inhibitor)*, 10–20 μM of AY9944 alone showed no effects on survival. When administered with LPS, the 10 μM dose led to partial protection against mortality, while 20 μM resulted in 100% survival up to 6 dpf. None of the other drugs tested protected against LPS death.

## Discussion

In this study, we performed an unbiased differential expression analysis of leukocyte gene expression RNA-seq data from diverse, prospective cohorts of sepsis patients. We further investigated 47 lipid metabolism genes to delineate lipid metabolic changes in sepsis patients by outcome and identified *DHCR7* to be significantly and consistently upregulated for patients with CCI/early death and in the 90-day mortality group, when compared to healthy controls and rapid recovery patients. *DHCR7*encodes an enzyme that removes the double bond in the B ring of sterols and catalyzes the conversion of 7-dehydroxycholesterol (7DHC) to cholesterol.^44^ 7DHC is also a precursor to vitamin D, catalyzed by *DHCR7*,^44^ In a parallel set of RNA-seq studies conducted in a zebrafish sepsis model, we observed that *dhcr7* was significantly upregulated in samples from zebrafish that received lethal doses of LPS when compared to controls. Moreover, pharmacologic blockade of Dhcr7 resulted in complete rescue from death. These results are consistent with *Dhcr7* having a potential mechanistic link to endotoxic death in a zebrafish sepsis model.

*DHCR7* is a critical gene involved in cholesterol biosynthesis, immune regulation and metabolism. Patients with loss of function mutations in *DHCR7* develop Smith-Lemli-Optiz syndrome, which results in branchial and cardiac defects, electrolyte abnormalities (hypocalcemia, hyponatremia, hyperkalemia) and extremely low cholesterol levels (< 38.7 mg/dL) associated with necrotizing enterocolitis, recurrent infections, sepsis-like episodes and death in several patients.^45^ In a recent study, the genetic association of variants in the *DHCR7* gene (and other genes for vitamin D metabolism) with subsequent bacterial pneumonia was studied.^46^ They found that genetic variants of *CYP2R1* but not *DHCR7, GEMIN2* or *HAL* were associated with increased risk of bacterial pneumonias.

Recently, the potential mechanistic role of *DHCR7* in combatting systemic infections has been studied. Xiao and colleagues showed that DHC7inhibition or genetic ablation enhanced both in vivo and in vitro macrophage-mediated anti-viral function.^47^ They demonstrated that two DHC7inhibitors (AY9944 and tamoxifen) led to increased clearance of vesicular stomatitis virus (VSV) and Zika virus. AY9944 administered to virus-infected (VSV or murine cytomegalovirus) macrophages led to enhanced *Ifnb* production in control macrophages but failed to enhance *Ifnb* production in *DHCR7*-deficient macrophages. The treatment of macrophages with tamoxifen also resulted in enhanced *Ifnb* expression upon treatment with a TLR3 agonist or VSV. Tamoxifen has also been shown to enhance neutrophil-mediated phagocytosis and extracellular trap formation to clear bacteria and has been proposed as a potential agent for combatting multi-drug resistant gram-negative infections.^48,49^

We discovered a number of genes involved in the cholesterol synthesis pathway to be upregulated in sepsis patients when compared to healthy controls. While this could be a general response to reduced LDL-C and HDL-C levels in sepsis, the expression of some of these genes discriminated sepsis patients with CCI/early death outcomes from those in the rapid recovery and control groups, suggesting potential bedside prognostic utility. Our mortality analysis also revealed some additional insights. The upregulation of *DHCR7* and PLTPfor 90-day mortality emphasizes the important role that *DHCR7* (and *PLTP)* may play in death from sepsis. In addition to regulating HDL size and facilitating cholesterol and LPS clearance, *PLTP* is critical to the immunomodulatory action of HDL and is a key factor in maintaining plasma sphingosine-1-phosphate levels (S1P).^23^ S1P which is primarily carried on HDL in association with apolipoprotein M, has antiapoptotic and chemotactic effects and levels decline in sepsis. Declining S1P levels have a strong inverse relationship with organ failure.^50^

This study had several limitations. First, this was a small prospective study of gene expression from a single center. Findings from this analysis should be confirmed in a larger and multi-center study. However, to increase the generalizability of our results, we used a diverse cohort of patients (gender and race) and derived and validated our results in two separate cohorts. Our initial RNA-seq analysis involved single-end sequencing, whereas the validation involved paired-end sequencing. This difference was due to technical advances in the Department of Pathology that sequenced our samples but should not affect interpretation of our results. Though our LPS zebrafish model of sepsis is a sterile model, we were able to recapitulate several aspects of human sepsis, namely mortality and similar differential expression patterns for the lipid metabolism genes of interest. Finally, being an observational study, there is no way to infer causality between observed gene expression differences and outcomes.

## Conclusion

In conclusion, this study identified *DHCR7* upregulation as potentially influencing poor outcomes after sepsis (CCI/early death) in humans. Our robust findings in human sepsis, confirmed in a validation cohort as well as with RT-qPCR analysis, were then recapitulated in a zebrafish LPS sepsis model with similar differential expression of *DHCR7* in LPS-treated zebrafish. Blockade of *Dhcr7* led to complete rescue of LPS-treated zebrafish from LPS death and may lead to therapeutic opportunities and drug repurposing for sepsis. These findings should be validated in larger, multi-center studies.

## Figures and Tables

**Table 1:** Demographic Features, Presenting Vital Signs, and Presenting Lipid Levels by Outcome.

Variable:		All patients (N = 128)	Rapid Recovery (N = 89)	CCI (N = 21)	Early Death (N = 18)	P Value
**Demographic Features:**						
Age (years): median [IQR)		61.5 [56.0, 70.0]	60.0 [54.0, 66.0]	72.0 [65.0, 78.0]	61.5 [57.0, 65.8]	0.036^b^
Gender: n (%)	Male	76 (59%)	59 (66%)	9 (43%)	8 (44%)	1^c^
	Female	52 (41 %)	30 (34%)	12 (57%)	10 (56%)	
Race: n (%)	African American	71 (55%)	49 (55%)	13 (62%)	9 (50%)	1^c^
	Caucasian	54 (42%)	38 (43%)	7 (33%)	9 (50%)	
	Other	3 (2%)	2 (2%)	1 (5%)	0 (0%)	
**Presenting Vital Signs:**						
Systolic blood pressure (mm Hg): median [IQR)		112.0 [97.0, 129.8]	115.0 [103.0, 129.0]	106.0 [92.0, 136.0]	102.5 [87.5, 124.5]	1^a^
Diastolic blood pressure (mm Hg): median [IQR)		61.0 [53.0, 74.0]	61.0 [54.0, 74.0]	65.0 [53.0, 77.0]	60.5 [44.8, 69.8]	1^a^
Heart rate (beats/min): median [IQR)		97.0 [84.0, 117.5] (1 missing)	97.0 [86.0, 116.5] (1 missing)	88.0 [73.0, 101.0] (1 missing)	106.0 [90.5, 121.5]	1^a^
**Demographic Features:**						
Respiratory rate (breaths/min): median [IQR)		19.0 [17.0, 22.5] (1 missing)	19.0 [16.0, 22.0]	20.0 [16.0, 24.0]	22.0 [19.0, 26.0] (1 missing)	1^a^
Temperature (F): median [IQR)		99.0 [98.1, 100.5] (1 missing)	99.1 [98.1, 100.7] (1 missing)	98.4 [97.7, 99.3]	99.3 [98.4, 100.2]	1^a^
Oxygen saturation (%): median [IQR)		98.0 [95.0, 99.0] (1 missing)	98.0 [95.0, 99.0]	98.0 [96.0, 99.3] (1 missing)	97.0 [95.0, 99.0]	1^a^
**Presenting Lipid Levels:**						
HDL (mg/dl): median [IQR)		21.7 [13.1, 34.6]	21.0 [13.8, 34.5]	18.1 [10.0, 30.0]	26.0 [14.7, 35.8]	1^a^
LDL (mg/dl): median [IQR)		58.5 [29.3, 84.8] (2 missing)	67.0 [47.0, 99.0]	27.0 [21.0, 61.1]	26.5 [17.8, 45.3] (2 missing)	<0.001^a^
Triglycerides (mg/dl): median [IQR)		99.5 [58.8, 142.3]	95.5 [47.0, 131.0]	116.0 [65.0, 144.0]	99.0 [69.8, 153.0]	1^a^
**Demographic Features:**						
Total cholesterol (mg/dl): median [IQR)		90.9 [74.0, 122.3]	102.0 [79.8, 125.0]	75.0 [65.0, 120.0]	76.5 [70.3, 95.3]	0.709^a^

a: Kruskal-Wallis Test

b: One-Way ANOVA

c: Fisher’s Exact Test

**Table 2:** Clinical Features and Management by Outcome.

Variable:		All patients (N = 128)	Rapid Recovery (N = 89)	CCI (N = 21)	Early Death (N = 18)	P Value
**Clinical Features:**						
T0 SOFA score: median [IQR)		6.0 [4.0, 10.0]	5.0 [3.0, 8.0]	11.0 [8.0, 11.0]	10.0 [6.5, 13.0]	<0.001^a^
Apache II score: median [IQR)		17.0 [11.0, 21.0] (39 missing)	13.0 [9.0, 18.0] (32 missing)	18.0 [14.8, 29.0] (5 missing)	21.0 [17.8, 25.0] (2 missing)	0.010^a^
Vasopressor use (0/1): n (%)		57 (45%)	25 (28%)	15 (71%)	17 (94%)	<0.001^c^
Pressor duration* (hours): median [IQR)		41.0 [24.0, 66.0]	34.0 [17.2, 41.0]	43.0 [26.1, 97.5]	62.1 [44.0, 128.8]	0.343^a^
Diabetes (0/1): n (%)		54 (42%)	38 (43%)	8 (38%)	8 (44%)	1^c^
COPD (0/1): n (%)		18 (14%)	11 (12%)	3 (14%)	4 (22%)	1^c^
ESRD (0/1): n (%)		15 (12%)	11 (12%)	3 (14%)	1 (6%)	1^c^
HIV (0/1): n (%)		4 (3%)	4 (4%)	0 (0%)	0 (0%)	1^c^
Primary Infection Source: n (%) (2 missing)	Blood without another source	5 (4%)	4 (4%)	0 (0%)	1 (6%)	
	Endocarditis	4 (3%)	2 (2%)	1 (5%)	1 (6%)	
	Intra-abdominal	11 (9%)	6 (7%)	2 (10%)	3 (18%)	
**Clinical Features:**						
	IV catheter-related bloodstream	2 (2%)	1 (1 %)	0 (0%)	1 (6%)	
	Necrotizing Soft Tissue	1 (1 %)	0 (0%)	1 (5%)	0 (0%)	
	Other	3 (2%)	3 (3%)	0 (0%)	0 (0%)	
	Pulmonary	39 (30%)	24 (27%)	10 (48%)	5 (28%)	
	Skin/soft tissue	17 (13%)	14 (16%)	2 (10%)	1 (6%)	
	Surgical Site	1 (1 %)	1 (1 %)	0 (0%)	0 (0%)	
	Surgical Thoracic	1 (1 %)	0 (0%)	1 (5%)	0 (0%)	
	Unknown	1 (1 %)	1 (1 %)	0 (0%)	0 (0%)	
	Urinary Tract	42 (33%)	33 (37%)	4 (19%)	5 (28%)	
**Treatment Variables:**						
Time to antibiotics from triage (minutes): median [IQR]		120.0 [76.5, 179.0] (1 missing)	122.0 [77.0, 180.0]	109.0 [67.0, 156.0]	134.0 [90.0, 190.0] (1 missing)	1^a^
Fluids volume in first 6 hours from triage (mL): median [IQR]		2000 [1000, 3137]	2000 [1000, 3000]	3000 [1500, 3330]	2500 [1000, 3500]	1^a^
Mechanical Vent Use (0/1): n (%)		46 (36%)	16 (18%)	15 (71%)	15 (83%)	<0.001^c^
**Clinical Features:**						
Mechanical Vent Duration (days): median [IQR] (2 missing)		2.9 [0.6, 9.5] (2 missing)	2.3 [0.7, 3.2]	7.8 [1.2, 19.4] (1 missing)	4.5 [0.5, 9.6] (1 missing)	1^a^
Length of hospital stay (days): median [IQR]		8.0 [4.9, 13.4]	7.1 [5.0, 12.9]	19.5 [12.0, 27.9]	6.3 [1.0, 9.0]	0.044^a^
ICU (0/1): n (%)		90 (70%)	52 (58%)	20 (95%)	18 (100%)	<0.001^c^
Length of ICU stay* (days): median [IQR]		5.0 [3.0, 11.0]	4.0 [2.0, 5.3]	17.0 [8.0, 29.0]	7.5 [3.3, 10.8]	<0.001^a^

* Statistical test and comparisons applied respectively for 57 patients on vasopressors and 90 patients in ICU

a: Kruskal-Wallis Test;

b: One-Way ANOVA;

c: Fisher’s Exact Test

## Data Availability

Supporting data from this study can be obtained by emailing the corresponding author Dr. Faheem W. Guirgis, MD.
